# Safety evaluation of saffron extracts in early and established atherosclerotic New Zealand white rabbits

**DOI:** 10.1371/journal.pone.0295212

**Published:** 2024-01-11

**Authors:** Iman Nabilah Abd Rahim, Noor Alicezah Mohd Kasim, Effat Omar, Suhaila Abdul Muid, Hapizah Nawawi

**Affiliations:** 1 Department of Pathology, Faculty of Medicine, Universiti Teknologi MARA, Sungai Buloh, Selangor, Malaysia; 2 Institute of Pathology, Laboratory and Forensic Medicine (I-PPerForM), Universiti Teknologi MARA, Sungai Buloh, Selangor, Malaysia; 3 Department of Biochemistry & Molecular Medicine, Faculty of Medicine, Universiti Teknologi MARA, Sungai Buloh, Selangor, Malaysia; North-Eastern Hill University, India, INDIA

## Abstract

Previous research has shown that natural medications pose health risks, especially in subjects with comorbidities. This study aimed to evaluate the safety of saffron ethanolic extract (SEE) administration in early and established atherosclerotic rabbits. Rabbits were given a high-cholesterol diet (HCD) for 4 and 8 weeks to induce early and established atherosclerosis respectively, and then they were treated with 50 and 100 mg/kg/day SEE. The body weight of the animals was recorded. Blood samples were collected at baseline, pre-treatment, and post-treatment for hematological studies, lipid profiles, and biochemical profiles. Tissue specimens of the vital organs were subjected to histological examination. The above parameters were significantly altered post-intervention with 4 and 8 weeks of HCD. No significant differences in body weight were observed in all the groups post-treatment with 50 and 100mg/kg of SEE compared to pre-treatment. However, low-density lipoprotein cholesterol, total cholesterol, serum urea, and glucose significantly decreased post-treatment with 50 and 100mg/kg/day SEE compared to pre-treatment in early and established atherosclerosis groups. Hematological parameters that were affected post-intervention with HCD returned to their baseline values post-treatment with 50 and 100mg/kg/day SEE. There was a significant improvement in the vital organs post-treatment with 50 and 100mg/kg SEE. SEE can safely be administered without causing harmful effects on the hematological, biochemical profiles, and vital organs. Notably, SEE exerts hypolipidemic and hypoglycemic effects on atherosclerotic conditions. Further clinical trials are warranted to ensure the safety of saffron administration in patients with atherosclerosis-related diseases.

## Introduction

It is estimated that 80% of the world’s population, which is up to four billion people rely on traditional medicine for some part of primary healthcare [1]. Recently, herbal remedies have been widely used in the prevention and treatment of various health problems worldwide. As the global use of herbal remedies continues to grow, their safety and efficacy have become a public health concern. Herbal medicines have been widely embraced in developed countries because they are natural and therefore considered to be safer than allopathic medicines [2].

Although several health problems have been treated by using medicinal plants, research has shown that using some natural medications poses risks due to unknown toxicity and side effects [3, 4]. Therefore, scientific approaches need to be applied to the safety and effectiveness of traditional plants in managing ailments. The World Health Organization emphasizes the importance of scientific investigations into indigenous herbal medicines and incorporated toxicological studies as part of the safety assessment of herbal medicines [4]. Besides, the effects and safety of herbal medicines on individuals with comorbidities need to be highlighted. Those with comorbidities should exercise caution since they are more susceptible to the side effects and toxicities of herbal medications.

Saffron, the dried stigma of the flower *Crocus sativus* L., is used as a food additive and considered a valued product for its yellowish color, bitter taste, and unique aroma. In addition to its use in gastronomy, saffron has long been considered a medicinal plant given its therapeutic properties in gynecological disorders, ocular disorders, mental disorders, respiratory problems, and digestive disorders [5].

Presently, saffron stigma extracts and its major bioactive compounds, crocin, crocetin, safranal, and picrocrocin are well-established to produce a variety of pharmacological and therapeutic effects on cardiovascular diseases [6–8], asthma [9, 10], depression [11, 12], dementia [13], premenstrual syndrome [14, 15], obesity [16], and diabetes[17]. Although most studies were performed in animals, small-scale clinical trials have also been reported in recent years [18–20].

Several *in vitro* and *in vivo* studies on the toxicity of saffron extract revealed that the LD50 value of saffron aqueous extract was 4120±556 mg/kg after oral administration in BALB/c mice [21]. In a subacute toxicity study of saffron ethanolic extract, histopathological findings depicted that 1.05 g/kg of the extract induced mild to severe hepatic and renal injuries in extract-treated rats [22]. Moreover, high doses of saffron ethanolic extract up to 4000 and 5000 mg/kg administration in a sub-chronic exposure results in significant changes in the serum biochemical parameters of mice, which were confirmed by the histopathological findings [23]. Nevertheless, a review on the toxicity of saffron reported that therapeutic doses of saffron exhibit no significant toxicity in both clinical and experimental investigations [24].

Despite the wide range of studies on the toxicity of saffron extract, no studies have investigated the effect of saffron on individuals with comorbidities, such as atherosclerosis. Besides, the safety of saffron extract remains unclear due to differences in the composition of various saffron grades, quality, and the method of sample preparation. Therefore, to ensure the safe use of saffron extracts in atherosclerotic conditions, the present study aims to investigate the safety of 50 and 100 mg/kg/day saffron ethanolic extract (SEE) administration in high-cholesterol diet (HCD) induced early and established atherosclerotic rabbits.

## Materials and methods

### Preparation of saffron ethanolic extract

The saffron (stigma of *Crocus sativus* L. flower) was purchased from Saharkhiz Saffron Co. (Mashhad, Iran). The voucher specimen was deposited at the herbarium, Faculty of Science and Technology, The National University of Malaysia. The species were confirmed as *Crocus sativus L*. with voucher specimen number ID006/2021. The maceration method was used to extract the stigma of the saffron plant. Briefly, 100 grams of dried-ground saffron stigma was soaked in 1500 mL of ethanol (80% v/v) with a magnetic stirrer for three days at room temperature. The mixture was then filtered and concentrated under reduced pressure at 40°C. The ethanol was removed using a rotary evaporator. The resulting extract was later kept at -80°C overnight and lyophilized using a freeze dryer. Lastly, the lyophilized extract was kept at -80°C until further use. The yield of the extract obtained was 50% (w/w). Chromatographic identification of bioactive compounds in a saffron extract from a previous study found that 1 gram sample of the dried saffron extract contained 290 mg and 19.13 mg of crocin and safranal, respectively [25].

### Animals and diets

Three to four months old male New Zealand White rabbits (NZWR), weighing 1.8 to 2.0 kg were purchased from A Sapphire Enterprise (Seri Kembangan, Malaysia). Only healthy animals were used in the study after two-week acclimation. Animals were caged individually in an environment-controlled clean air room with a temperature of 22 ± 2°C, 12 h light/ 12 h dark cycle with a relative humidity of 60 ± 5% at the Laboratory Animal Care Unit (LACU) of Universiti Teknologi MARA (UiTM). The animals were provided with a sterilized laboratory rabbit diet and autoclaved water ad libitum. All experimental procedures were performed during the light cycle in a separate laboratory and an appropriate animal experimentation facility. The animal experiments in this study were approved by and conducted in conformity with the rules and regulations of the Universiti Teknologi MARA Committee on Animal Research & Ethics (UiTM CARE) with an ethical approval number of UiTM CARE: 326/2020.

### Induction of atherosclerosis and experimental design

**Fig 1:**
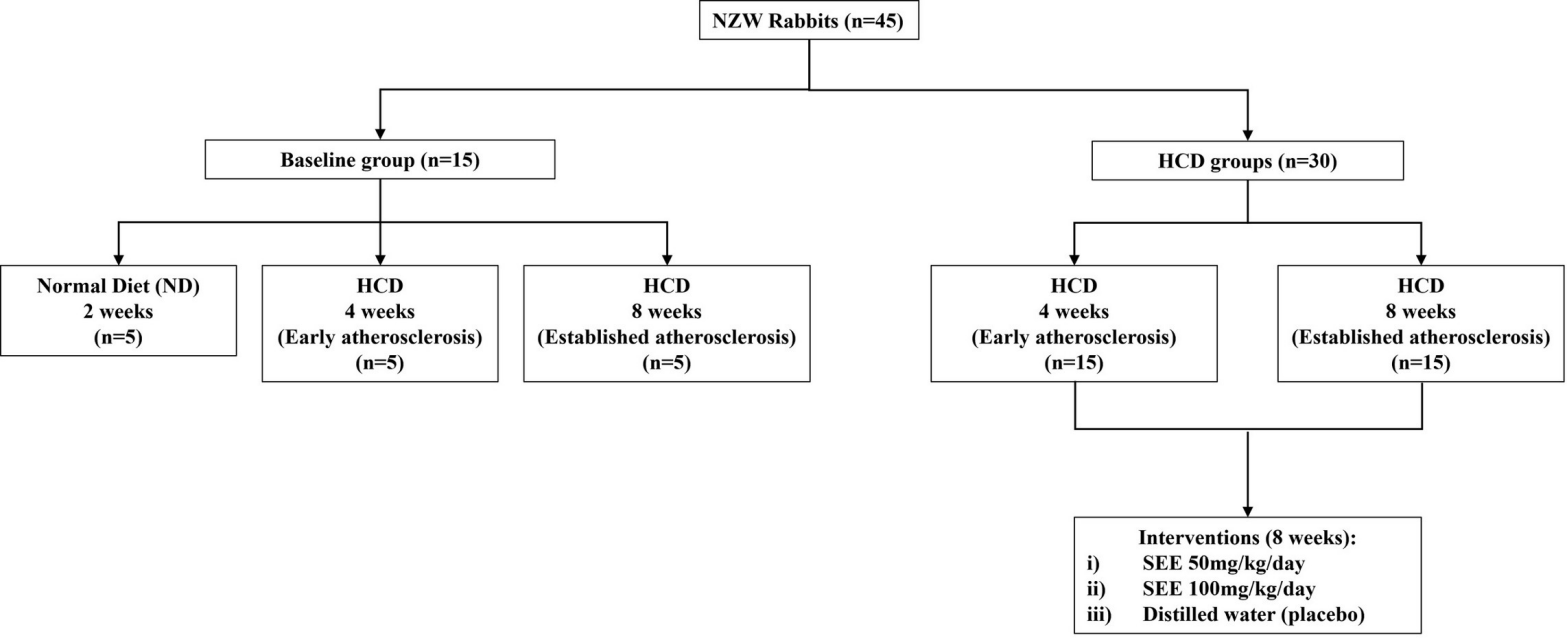
Experimental design of the study.

After 2 weeks of acclimatization, 45 NZWR were randomly assigned into 2 groups: baseline group (n = 15) and treatment group (n = 30). In the baseline group, rabbits were divided into 3 groups: (1) given a normal diet (ND) (n = 5) for 2 weeks, (2) fed with 50g/kg/day 1% high-cholesterol diet (HCD) for 4 weeks (n = 5) to induce early atherosclerosis and (3) fed with 50g/kg/day 1% HCD for 8 weeks (n = 5) to induce established atherosclerosis. The baseline group was used as the tissue control. Rabbits in the treatment group were divided into 2 groups. They were administered with the same amount and percentage of HCD as above for 4 and 8 weeks to induce early and established atherosclerosis, respectively (pre-treatment group). The animals were then further divided into 3 groups (n = 5 each): group I: 50mg/kg/day saffron ethanolic extract (SEE) (S50), group II: 100mg/kg/day SEE (S100) and group III: distilled water (placebo). The dose was selected based on a previous study that utilized mice for their experiment [26]. Subsequently, the dose was converted to a rabbit dosage using the conversion formula by Nair et al. (2016) [27]. Rabbits were given ND during the treatment period. Both doses of SEE were forced-fed to the rabbits daily for 8 weeks. The rabbits were gently wrapped in a blanket during force-feeding, leaving only the head exposed. To prevent aspiration, the syringe was filled with the appropriate dose of SEE and examined for any air bubbles. The syringe was then placed between the front and back teeth to ensure that all the extracts were fully ingested by the rabbits. Accurate dosage or food portion was ensured by measuring daily food intake. SEE was reset based on the changes in weekly body weight. The experimental protocol was depicted in Fig 1.

### Body weight measurement

Body weight (g) was determined in the rabbits at the baseline (week 2), post-HCD feeding (weeks 6 and 10 in the early and established atherosclerosis group, respectively), and post-treatment (weeks 14 and 18 in the early and established atherosclerosis group, respectively) of the experimental period. An electronic digital scale (model EK3350-31P, Camry, China) with a maximum capacity of 5000 g and a minimum of 1 g was used for measurement.

### Analysis of blood and serum biochemistry

Whole blood was collected at three different intervals; baseline, post-HCD feeding (or pre-treatment), and post-treatment from the marginal ear vein or the central auricular artery and used for hematological and serum biochemical analysis. A total of 8 ml blood was collected in ethylenediaminetetraacetic acid (EDTA) tube and used for measurement of total and differential cell counts, white blood cells (WBC), red blood cells (RBC), hemoglobin (HGB), hematocrit (HCT), mean corpuscular volume (MCV), mean corpuscular hemoglobin (MCH), mean corpuscular hemoglobin concentration (MCHC), platelet, neutrophil, lymphocyte, monocyte, eosinophil and basophil indices using a Sysmex XN-550 automated blood cell counter. Serum was separated by centrifugation at 4000 rpm for 10 mins and stored at −80°C until analysis. The serum was analyzed for lipid profile test such as total cholesterol (TC), triglycerides (TG), low-density lipoprotein cholesterol (LDL) and high-density lipoprotein cholesterol (HDL), liver function test such as alkaline phosphatase (ALP), aspartate aminotransferase (AST), alanine aminotransferase (ALT) and gamma-glutamyl transferase (GGT), renal function test such as creatinine and urea, and glucose using an automated analyzer Roche Cobas c501.

### Histopathological analysis

All major organs such as the liver, kidney, brain, spleen, heart and lungs of the rabbits were excised immediately after gross examination and fixed in 10% neutral buffered formalin. Sufficiently fixed organs were embedded in paraffin after dehydration in graded ethanol and xylene and further stained with hematoxylin and eosin after cutting into sections of about 3–4 μm thickness. Histopathological evaluation was performed by a pathology specialist.

### Statistical analysis

The normal distribution of data was investigated using the Shapiro-Wilk test. All values were expressed as mean ± standard error of the mean (SEM). Body weight and laboratory results were compared within groups using the Paired samples t-test. Results between the groups were compared using the one-way analysis of variance (ANOVA), followed by the Bonferroni post hoc test. Statistical analysis was performed in SPSS software (IBM) version 27 and a *p*-value less than 0.05 was considered statistically significant.

## Results

### Body weight

**Fig 2:**
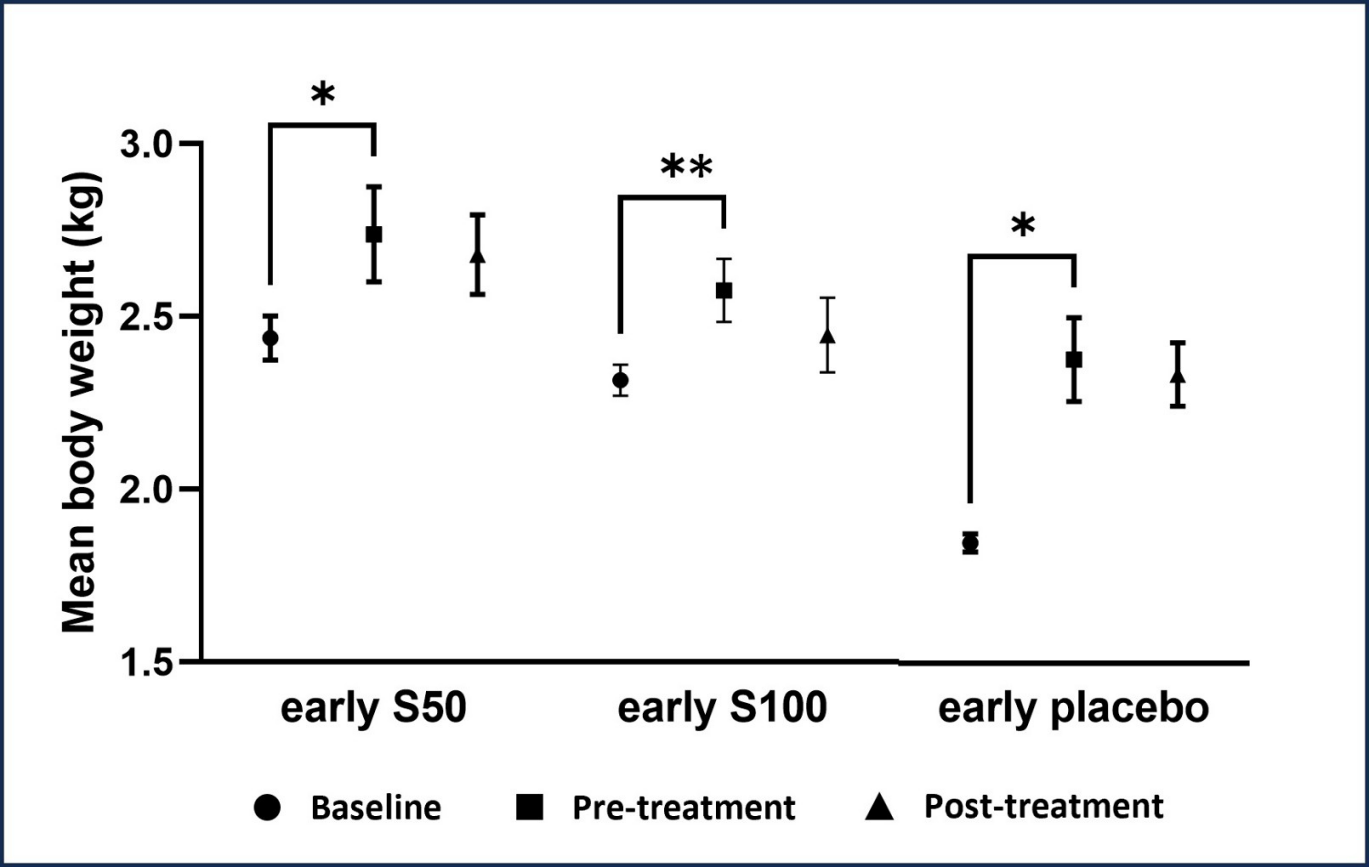
Effects of SEE and placebo on body weight of NZWR in early atherosclerosis group. Data are represented as mean ± SEM (n = 5). Significance from baseline is represented as: * at *p*<0.05 (significant) and **p<0.01 (highly significant).

**Fig 3:**
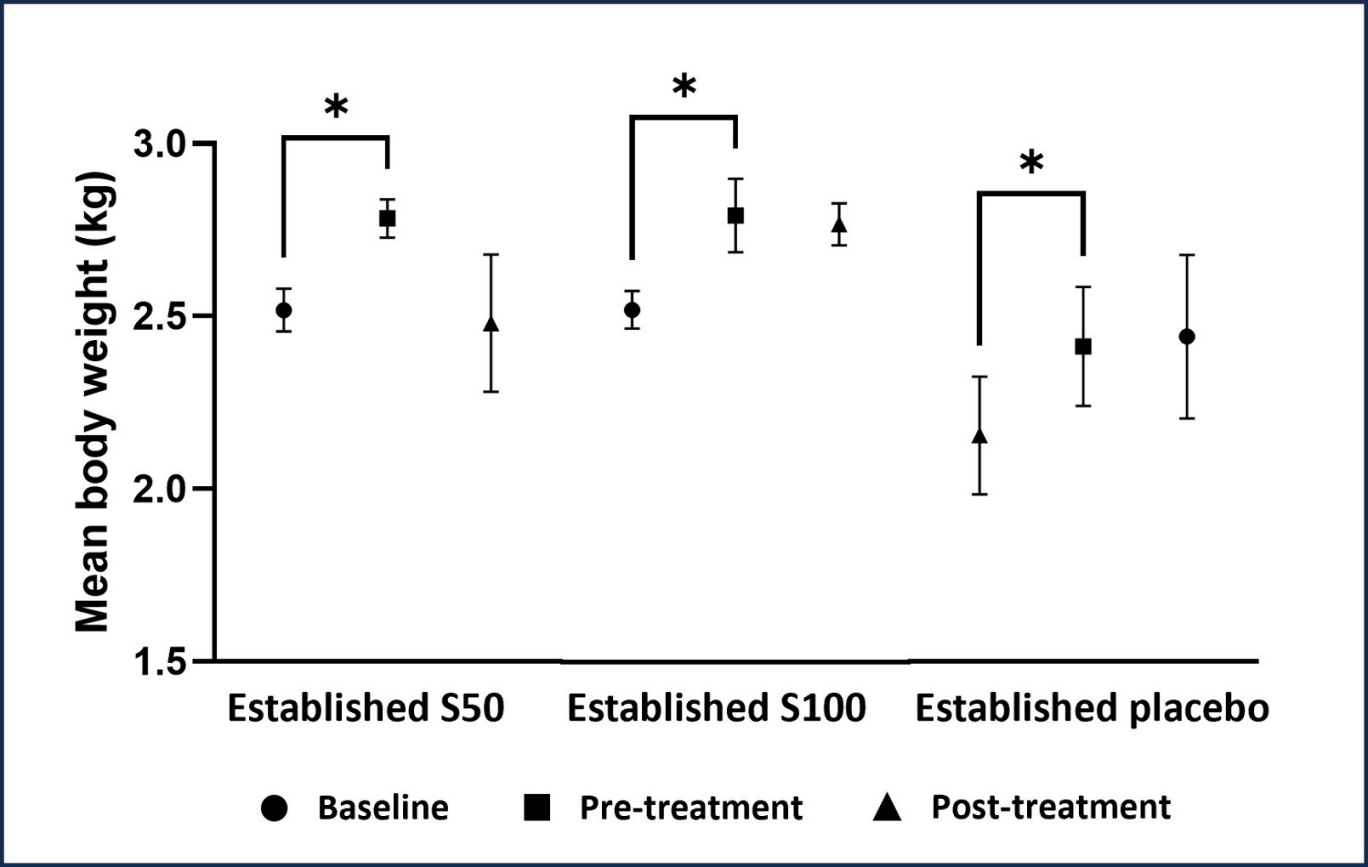
Effect of SEE and placebo on body weight of NZWR in established atherosclerosis group. Data are represented as mean ± SEM (n = 5). Significance from baseline is represented as: * at *p*<0.05 (significant).

The body weight of NZWR significantly increased after administering HCD for 4 (Fig 2) and 8 weeks (Fig 3) compared to baseline in all groups (*p* < 0.05). However, 50 and 100mg/kg of SEE and placebo in early and established atherosclerosis groups depicted no significant effect on the body weight of NZWR all over the experimental period compared to pre-treatment (*p* > 0.05).

### Biochemical analysis

#### Lipid profile

**Fig 4:**
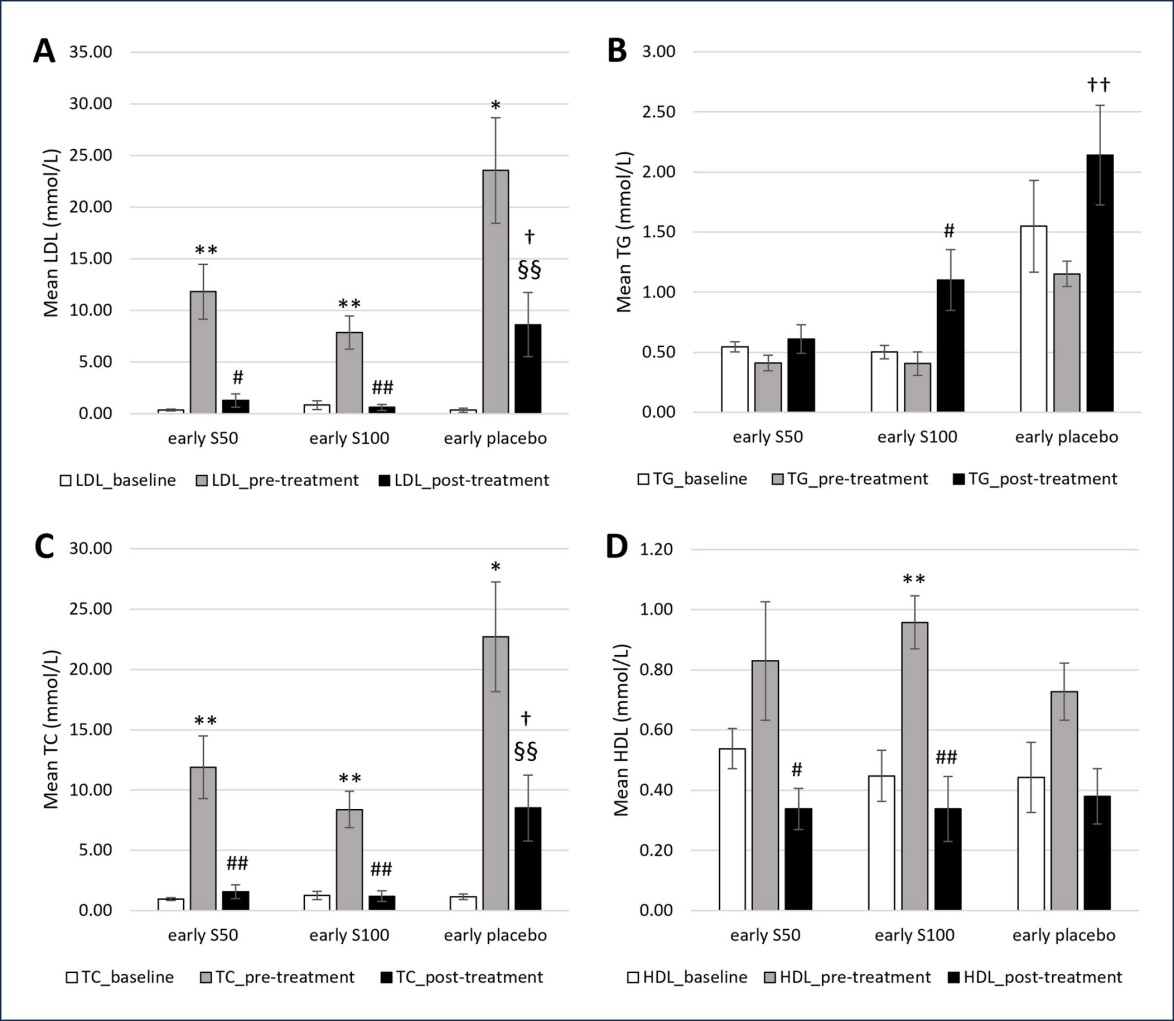
A-D. Serum lipid profile levels of rabbits at baseline, pre-treatment, and post treatment with SEE and placebo in early atherosclerosis group. * significant differences (*p*<0.05) vs baseline while** highly significant differences (*p*<0.01) vs baseline; # significant differences (*p*<0.05) vs pre-treatment while ## highly significant differences (*p*<0.01) vs pre-treatment. † significant differences (*p*<0.05) vs S50 while †† highly significant differences (*p*<0.01) vs S50. § significant differences (*p*<0.05) vs S100 while §§ highly significant differences (*p*<0.01) vs S100.

**Fig 5:**
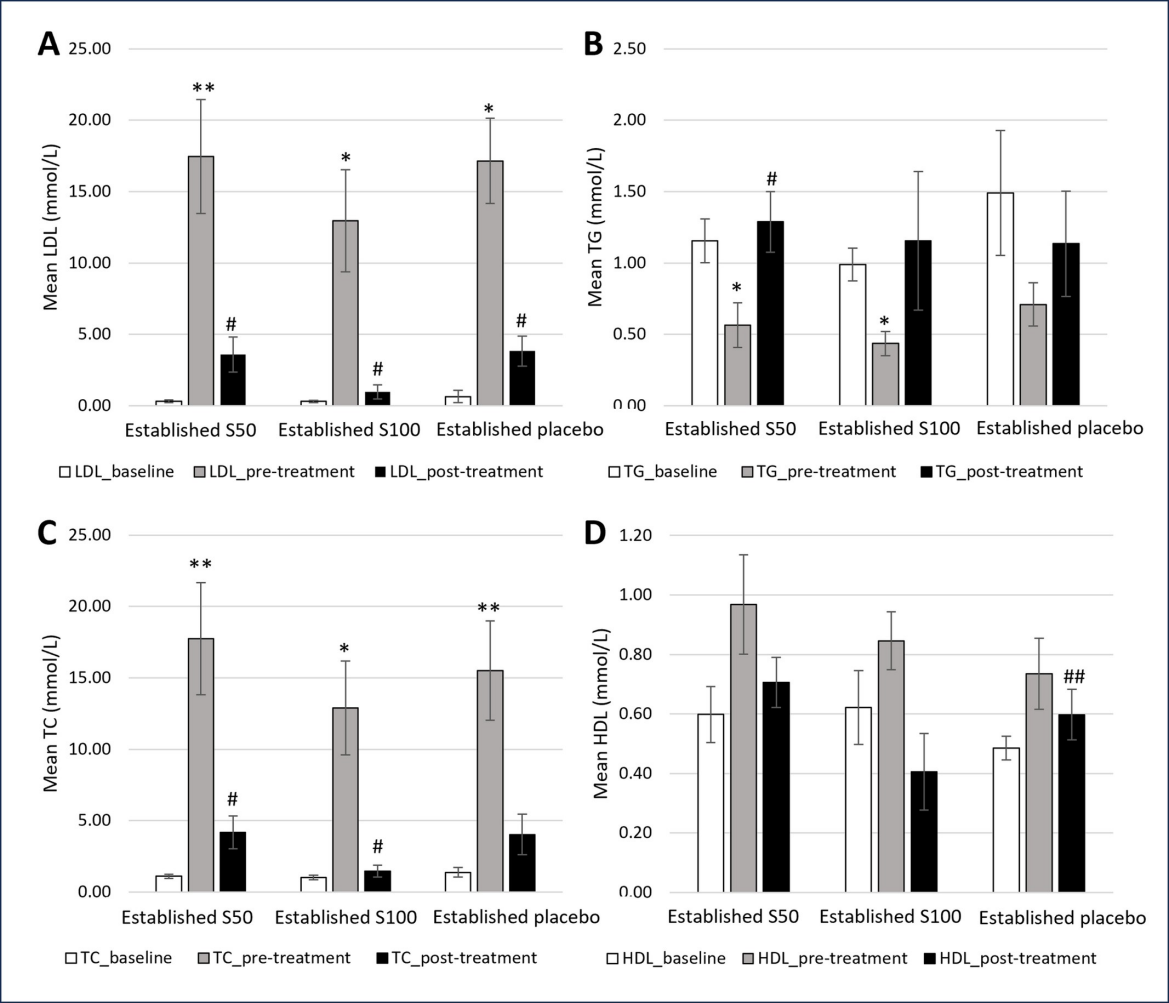
A-D. Serum lipid profile levels of rabbits at baseline, pre-treatment, and post treatment with SEE and placebo in established atherosclerosis group. * significant differences (*p*<0.05) vs baseline while ** highly significant differences (*p*<0.01) vs baseline. # significant differences (*p*<0.05) vs pre-treatment while ## highly significant differences (*p*<0.01) vs pre-treatment.

Figs 4A–4D and 5A–5D summarises the plasma TG, TC, LDL, and HDL in early and established atherosclerosis groups, respectively. LDL and TC significantly increased post-intervention with 4 and 8 weeks of HCD in all groups. In the early atherosclerosis group, HDL markedly increased post-intervention with HCD. In the established atherosclerosis group, TG significantly decreased in S50 and S100 groups compared to baseline.

In contrast to the above effects, LDL and TC significantly decreased post-treatment with 50 and 100mg/kg SEE compared to pre-treatment in early and established atherosclerosis groups. In the early atherosclerosis group, TG significantly increased in the S100 group while HDL markedly decreased in S50 and S100 groups. TG significantly increased in the S50 group, whereas HDL decreased in the S100 group in the established atherosclerosis group.

#### Liver and renal function test

The results revealed that four weeks of consumption of 50g/kg of 1% HCD had no significant effect on all the serum biochemical markers except for serum urea and ALT. Serum urea markedly decreased compared to baseline in the S50 group and significantly increased compared to baseline in the placebo group. Serum ALT markedly decreased in the placebo group compared to baseline (Table 1). However, eight weeks of HCD consumption results in significant changes in several serum biochemical markers; serum urea, AST, and glucose. Serum creatinine, ALP, ALT, and GGT did not show any prominent effects compared to the basal value (Table 2).

**Table 1:** Biochemical analysis of rabbits in early atherosclerosis group.

Biochemical parameters	S50			S100			Placebo		
	Baseline	Pre-treatment	Post-treatment	Baseline	Pre-treatment	Post-treatment	Baseline	Pre-treatment	Post-treatment
ALT (U/L)	50.4±2.9	43.2±8.2	38.4±3.3	39.0±8.7	46.8±13.4	35.9±5.3	55.7±6.8	12.8±3.5*	23.7±4.0
AST (U/L)	32.5±4.6	50.9±10.05	48.4±9.4	33.7±13.6	51.9±11.6	41.1±4.4	37.9±8.4	23.0±9.9	12.5±2.7^††§^
ALP (U/L)	100.8±21.4	84.4±11.3	61.2±9.4	71.2±6.7	72.8±6.8	57.0±10.6	143.5±24.4	113.8±20.8	122.5±35.5
GGT (U/L)	7.8±1.5	8.2±2.7	5.8±1.4	6.4±0.7	6.0±1.0	5.2±0.7	8.8±1.3	8.8±1.3	9.3±3.4
Urea (mmol/L)	8.5±0.7	6.5±0.3*	4.5±0.2^##^	7.7±1.0	6.1±0.3	4.8±0.2^#^	4.7±0.1^††^^§^	9.0±1.1*s^†^^§^	5.7±0.8^##^
Creatinine (μmol/L)	92±8.7	81.5±3.3	101.3±7.9	91.6±9.6	79±3.8	95.6±5.9	120.8±9.5	133±14.8^††^^§§^	114.3±25.0
Glucose (mmol/L)	10.2±1.6	10.3±0.7	6.5±0.2^##^	8.1±0.3	9.6±0.7	6.9±0.2^#^	8.6±0.2	8.5±0.7	7.5±0.4^†^

Values are means ± SEM; number of animals per group = 5.

*: p<0.05

**: p <0.01 significantly different compared to baseline.

#: p <0.05

##: p <0.01 significantly different compared to pre-treatment.

†: p<0.05

††: p<0.01 significantly different compared to S50.

§p<0.05

§§p<0.01 significantly different compared to S100.

**Table 2:** Biochemical analysis of rabbits in established atherosclerosis group.

Biochemical parameters	S50			S100			Placebo		
	Baseline	Pre-treatment	Post-treatment	Baseline	Pre-treatment	Post-treatment	Baseline	Pre-treatment	Post-treatment
ALT (U/L)	22.1±2.3	22.5±3.5	24.4±1.9	21.5±1.7	19.9±1.6	27.2±0.9^##^	44.7±1.4^††^^§§^	21.3±5.1	28.0±3.4
AST (U/L)	9.9±2.4	50.4±9.9*	34.6±2.4	8.8±1.2	37.5±5.6**	43.1±8.4	42.3±10.5^††^^§§^	25.5±2.5	63.3±26.7
ALP (U/L)	113.6±14.5	72.8±15.4	52.0±12.2^§§^	149.7±14.3	88.3±9.8	159.7±12.8^††^	76.0±4.2^§^	52.0±4.4	48.3±5.5^§§^
GGT (U/L)	5.2±0.9	7.0±0.9	7.8±0.4	9.4±1.9	8.8±1.4	10.0±1.6	7.4±1.2	8.0±1.3	8.2±2.5
Urea (mmol/L)	6.3±0.4	9.8±0.2**	5.3±0.5^##^	6.0±0.4	9.2±0.4**	4.9±0.4^##^	7.3±0.5	7.6±0.9	9.2±3.2
Creatinine (μmol/L)	83.2±7.5	86.6±6.2	77.8±8.9	88.2±7.6	93.6±6.6	97.2±7.2	102.0±1.0	114.3±4.4	84.0±10.5
Glucose (mmol/L)	8.8±0.5	10.3±0.6*	6.9±0.2^#^	10.1±1.1	10.9±0.6	6.6±0.1^##^	9.6±0.7	11.2±0.8*	7.3±0.5^#^

Values are means ± SEM; number of animals per group = 5.

*: p<0.05

**: p <0.01 significantly different compared to baseline.

#: p <0.05

##: p <0.01 significantly different compared to pre-treatment.

†: p<0.05

††: p<0.01 significantly different compared to S50.

§p<0.05

§§p<0.01 significantly different compared to S100.

In the early atherosclerosis group, daily oral administration of both doses of SEE and placebo for 8 consecutive weeks to NZWR reflected insignificant effects on some biochemical profiles, such as serum creatinine, AST, ALP, ALT, and GGT compared to pre-treatment. There were marked decrease in serum glucose and serum urea post-treatment with 50 and 100 mg/kg/day SEE compared to pre-treatment. However, only serum urea significantly decreased in the placebo group compared to pre-treatment (Table 1).

Meanwhile, in the established atherosclerosis group, both doses of SEE and placebo did not affect the serum activity of creatinine, AST, ALP, and GGT as compared to before treatment. Post-treatment with 100 mg/kg/day of SEE induced an increase in the level of serum ALT, while the levels of serum urea showed a significant reduction in NZWR administered 50 and 100 mg/kg/day SEE compared to pre-treatment. Serum glucose depicted a significant reduction in the S50, S100, and placebo groups compared to pre-treatment (Table 2).

### Hematological analysis

Post-intervention with 4 weeks HCD, MCH and MCHC significantly increased in S50, S100 and placebo groups compared to baseline. Neutrophils markedly decreased in S50 and S100 groups compared to the basal value (Table 3).

**Table 3:** Hematological analysis and percentage of WBC differential count of rabbits in early atherosclerosis group.

Hematological parameters	S50			S100			Placebo		
	Baseline	Pre-treatment	Post-treatment	Baseline	Pre-treatment	Post-treatment	Baseline	Pre-treatment	Post-treatment
WBC (10^3^/uL)	6.51±0.46	6.92±0.78	6.59±1.13	6.79±0.55	6.44±0.79	5.22±0.65	6.84±0.40	8.89±0.56	10.17±0.32
RBC (10^6^/uL)	5.37±0.17	4.63±0.44	6.54±0.30^##^	5.47±0.27	4.50±0.30	5.78±0.27	5.30±0.21	4.36±0.24	5.65±0.14^#^
HGB (g/dL)	11.76±0.33	10.66±0.99	14.42±0.50^##^^§^	11.93±0.68	10.38±1.23	12.08±0.74^†^	11.08±0.27	10.43±0.45	12.23±0.18^#†^
HCT (%)	36.64±0.81	31.54±2.91	43.26±1.28^##^^§§^	36.93±1.56	30.85±3.65	36.88±1.43^††^	33.98±0.83	30.68±1.27	38.25±0.57^##†^
MCV (fL)	68.30±0.97	68.26±0.56	66.40±1.30	67.63±0.94	68.60±0.57	63.93±1.18^#^	64.28±1.18	70.65±1.91	67.93±2.70
MCH (pg)	21.88±0.17	23.06±0.06**	22.12±0.31^#^	21.80±0.25	23.08±0.08**	20.85±0.55^#^	20.98±0.46	23.98±0.59*	21.70±0.83
MCHC (g/dL)	32.10±0.25	33.78±0.27**	33.32±0.42	32.28±0.48	33.63±0.28*	32.65±0.86	32.60±0.16	33.98±0.31**	31.95±0.16^##^
PLT (10^3^/ uL)	327.0±62.0	189.5±45.5	180.5±20.5	354.5±27.5	189.5±45.5	160.5±131.5	198.0±28.3	114.3±20.9	159.3±27.5
NEUT (%)	1.88±0.19	1.09±0.11**	1.66±0.36	2.04±0.33	1.04±0.13*	1.58±0.78	2.29±0.45	2.20±2.20^†^^§§^	2.56±0.32
LYMPH (%)	4.08±0.09	5.65±0.70	4.70±0.78	4.40±0.49	5.24±0.72	3.38±0.55	3.51±0.13	5.83±0.90	6.09±0.24
MONO (%)	0.38±0.16	0.11±0.02	0.13±0.06	0.22±0.12	0.11±0.02	0.15±0.07	0.20±0.06	0.21±0.02^†^^§§^	0.60±0.19^†^
EO (%)	0.0±0.0	0.0±0.0	0.0±0.0	0.0±0.0	0.0±0.0	0.0±0.0	0.0±0.0	0.0±0.0	0.0±0.0
BASO (%)	0.18±0.08	0.06±0.01	0.10±0.03	0.13±0.07	0.06±0.01	0.11±0.08	0.10±0.03	0.09±0.03	0.08±0.02

Values are mean ± SEM; number of animals per group = 5.

*: p<0.05

**: p <0.01 significantly different compared to baseline.

#: p <0.05

##: p <0.01 significantly different compared to pre-treatment.

†: p<0.05

††: p<0.01 significantly different compared to S50.

§p<0.05

§§p<0.01 significantly different compared to S100.

Post-intervention with 8 weeks HCD, MCH significantly increased in S50, S100 and placebo groups, while MCHC only increased in S100 group.Lymphocyte percentage markedly increased in S100 and placebo groups in comparison to the baseline (Table 4).

**Table 4:** Hematological analysis of rabbits and percentage of WBC differential count in established atherosclerosis group.

Hematological parameters	S50			S100			Placebo		
	Baseline	Pre-treatment	Post-treatment	Baseline	Pre-treatment	Post-treatment	Baseline	Pre-treatment	Post-treatment
WBC (10^3^/uL)	7.54±0.54	9.78±1.59	7.95±1.37	7.19±0.54	9.11±0.86	6.25±0.64^##^	7.50±0.93	7.98±1.29	6.72±0.30
RBC (10^6^/uL)	5.45±0.23	4.34±0.60	5.75±0.44	5.41±0.18	4.85±0.30	5.68±0.25	5.56±0.19	5.02±0.59	5.79±0.32
HGB (g/dL)	11.60±0.53	10.23±0.99	11.18±1.79	11.58±0.41	11.18±0.68	12.82±0.67	11.67±0.50	11.20±1.02	12.33±0.62
HCT (%)	35.50±1.51	30.35±2.71	36.95±1.79	34.47±1.56	33.67±2.11	38.07±1.41	36.03±1.44	32.83±2.52	36.90±1.27
MCV (fL)	65.18±1.69	71.53±3.80	64.70±2.41	65.86±1.48	67.46±0.88	70.10±1.32	64.80±2.42	66.03±2.81	63.93±1.39
MCH (pg)	21.30±0.60	24.03±1.24*	19.45±0.66^##^^§§^	21.44±0.49	23.08±0.29**	22.56±0.44^††^	21.00±0.72	22.47±0.61*	21.33±0.15
MCHC (g/dL)	32.65±0.16	33.63±0.50	30.10±0.86^#^	32.52±0.18	34.22±0.57*	32.22±0.53	32.33±0.18	34.03±0.56	33.40±0.55^†^
PLT (10^3^/ uL)	273.50±64.50	188.00±27.35	304.50±115.08	267.00±50.39	241.00±36.25	256.20±87.32	316.00±65.73	219.67±17.25	122.00±36.91
NEUT (%)	2.83±0.57	3.00±0.83	3.40±0.77	2.64±0.48	2.22±0.30	2.13±0.57	3.14±0.63	1.85±0.54	1.88±0.54
LYMPH (%)	4.24±0.35	6.30±1.17	3.92±1.56	4.09±0.31	6.36±0.76*	3.75±0.46^#^	4.24±0.41	6.30±0.57**	3.92±0.67
MONO (%)	0.25±0.12	0.30±0.15	0.42±0.12	0.27±0.10	0.41±0.12	0.25±0.07	0.25±0.10	0.30±0.16	0.42±0.20
EO (%)	0.0±0.0	0.0±0.0	0.0±0.0	0.0±0.0	0.0±0.0	0.0±0.0	0.0±0.0	0.0±0.0	0.0±0.0
BASO (%)	0.22±0.03	0.18±0.08	0.21±0.07	0.24±0.03	0.12±0.03	0.11±0.03	0.22±0.06	0.18±0.03	0.21±0.05

Values are mean ± SEM; number of animals per group = 5.

*: p<0.05

**: p <0.01 significantly different compared to baseline.

#: p <0.05

##: p <0.01 significantly different compared to pre-treatment.

†: p<0.05

††: p<0.01 significantly different compared to S50.

§p<0.05

§§p<0.01 significantly different compared to S100.

Post-treatment with 50 mg/kg/day of SEE and placebo, the hematological analysis of NZWR in the early atherosclerosis group reflected a significant increase in RBC, HGB, and HCT compared to pre-treatment. MCH decreased significantly in the S50 group while MCHC reduced post-treatment with placebo. A significant reduction of MCV and MCH was observed post-treatment with 100 mg/kg/day SEE compared to before treatment. Meanwhile, with 8 weeks of HCD consumption, MCH increased significantly in all the groups; S50, S100, and placebo, whereas MCHC and lymphocytes increased significantly in S100 and placebo groups.

In the established atherosclerosis group, MCH and MCHC markedly decreased post-treatment with 50 mg/kg/day SEE, while WBC and lymphocytes significantly decreased in the S100 group relative to before treatment.

### Histopathological analysis

**Fig 6:**
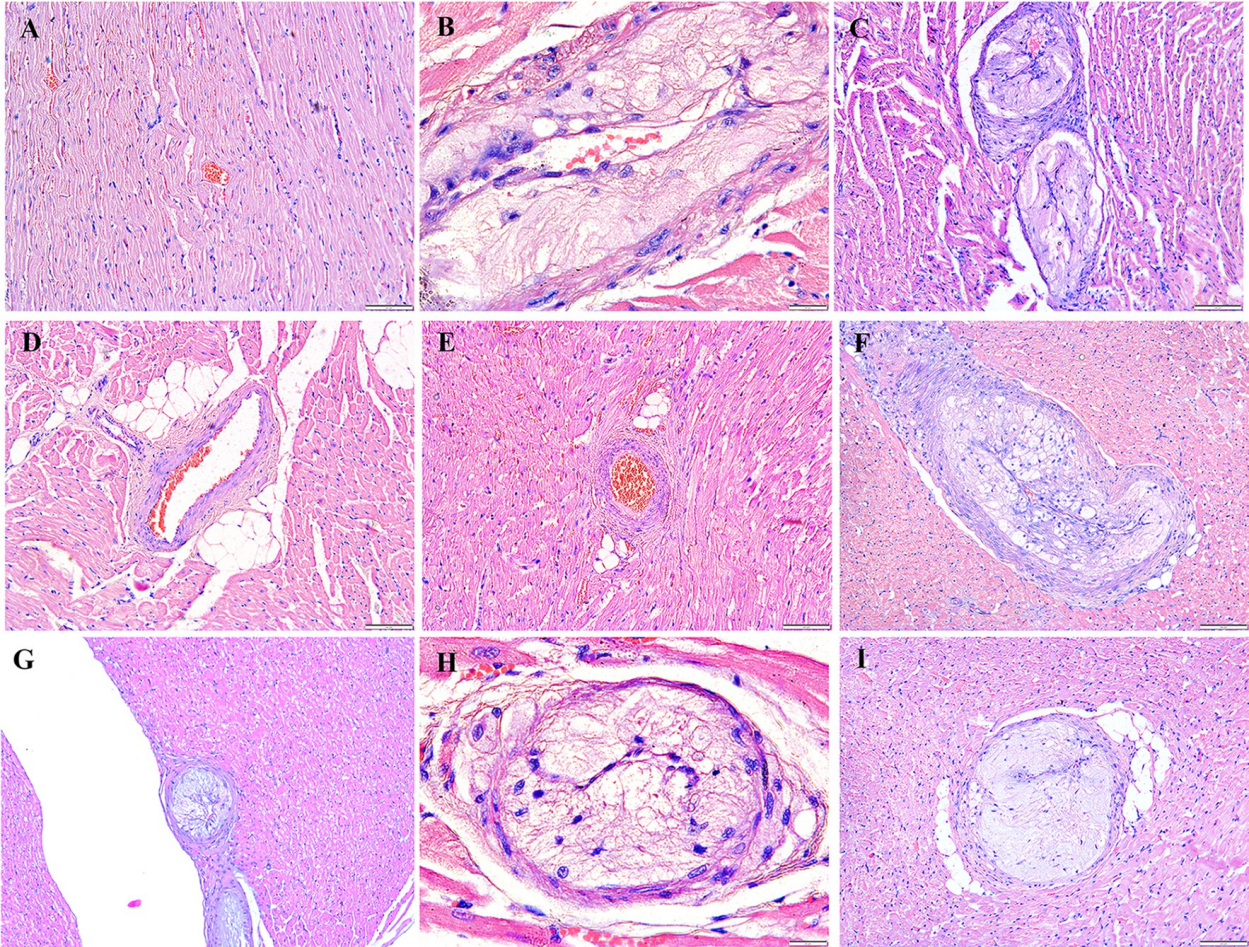
A-I. Photomicrograph of the heart stained with H&E (10x and 40x). A: Baseline group; B: 4 weeks HCD; C: 8 weeks HCD; D: Early atherosclerosis group S50; E: Early atherosclerosis group S100; F: Early atherosclerosis group placebo; G: Established atherosclerosis group placebo; H: Established atherosclerosis group S50; I: Established atherosclerosis group S100. Arrowhead shows thickening of blood vessels and formation of foam cells in the intimal layer.

**Fig 7:**
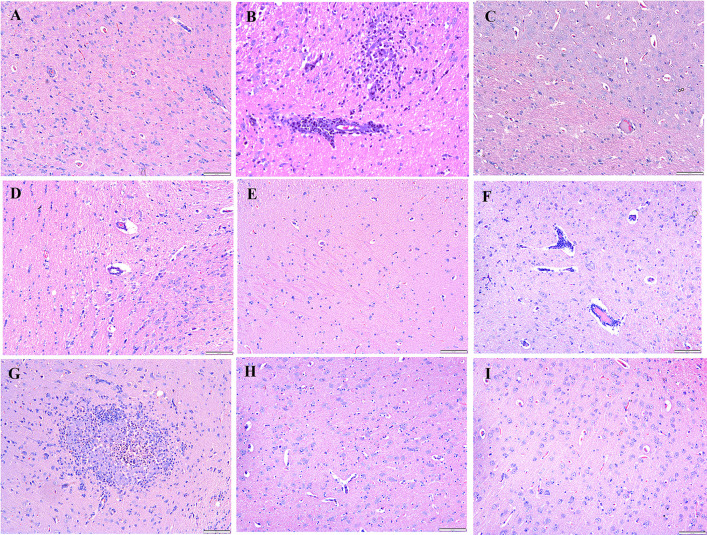
A-I. Photomicrograph of the brain stained with H&E (10x and 40x). A: Baseline group; B: 4 weeks HCD (arrowhead: perivascular lymphocytic cuffing); C: 8 weeks HCD; D: Early atherosclerosis group S50; E: Early atherosclerosis group S100; F: Early atherosclerosis group placebo (arrowhead: mild intraparenchymal lymphocyte aggregation); G: Established atherosclerosis group placebo (arrowhead: perivascular lymphocytic infiltration); H: Established atherosclerosis group S50; I: Established atherosclerosis group S100.

**Fig 8:**
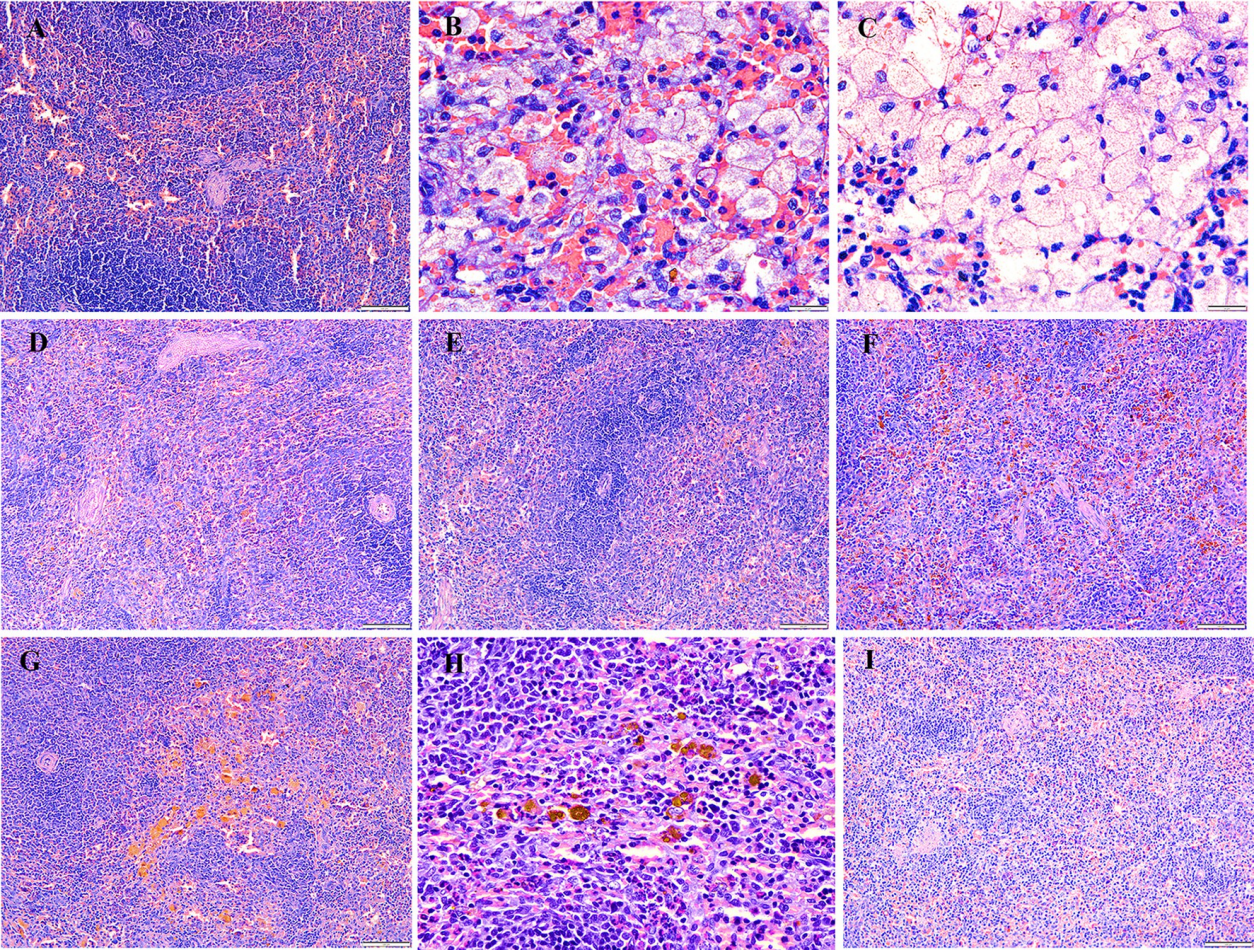
A-I. Photomicrograph of the spleen stained with H&E (10x and 40x). A: Baseline group; B: 4 weeks HCD; C: 8 weeks HCD; D: Early atherosclerosis group S50; E: Early atherosclerosis group S100; F: Early atherosclerosis group placebo; G: Established atherosclerosis group placebo; H: Established atherosclerosis group S50; I: Established atherosclerosis group S100. Arrowhead: Foam cells formation in the red pulp of spleen. Arrow: Hemosiderin-laden macrophages in the red pulp of spleen.

**Fig 9:**
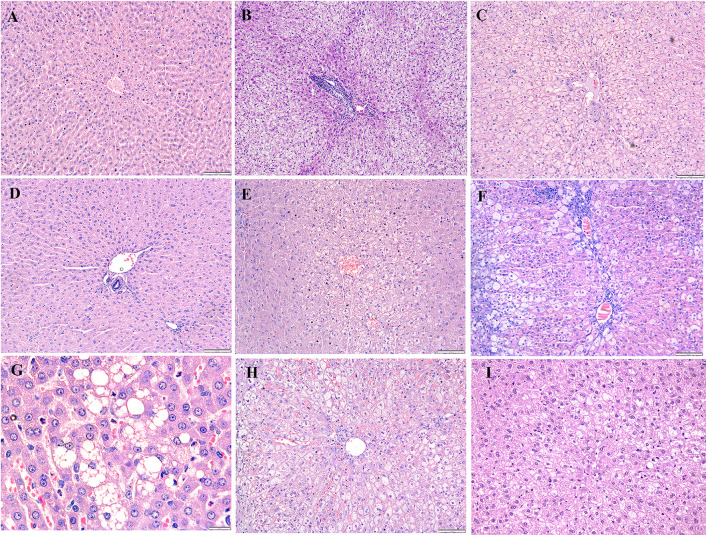
A-I. Photomicrograph of the liver stained with H&E (10x and 40x). A: Baseline group; B: 4 weeks HCD (arrow: portal inflammation and bridging inflammation with mild intralobular inflammation); C: 8 weeks HCD (arrowhead: steatosis of the liver); D: Early atherosclerosis group S50; E: Early atherosclerosis group S100; F: Early atherosclerosis group placebo (arrow: portal inflammation with severe intralobular inflammation); G: Established atherosclerosis group placebo (arrowhead: steatosis of the liver); H: Established atherosclerosis group S50; I: Established atherosclerosis group S100 (arrowhead: mild steatosis).

**Fig 10:**
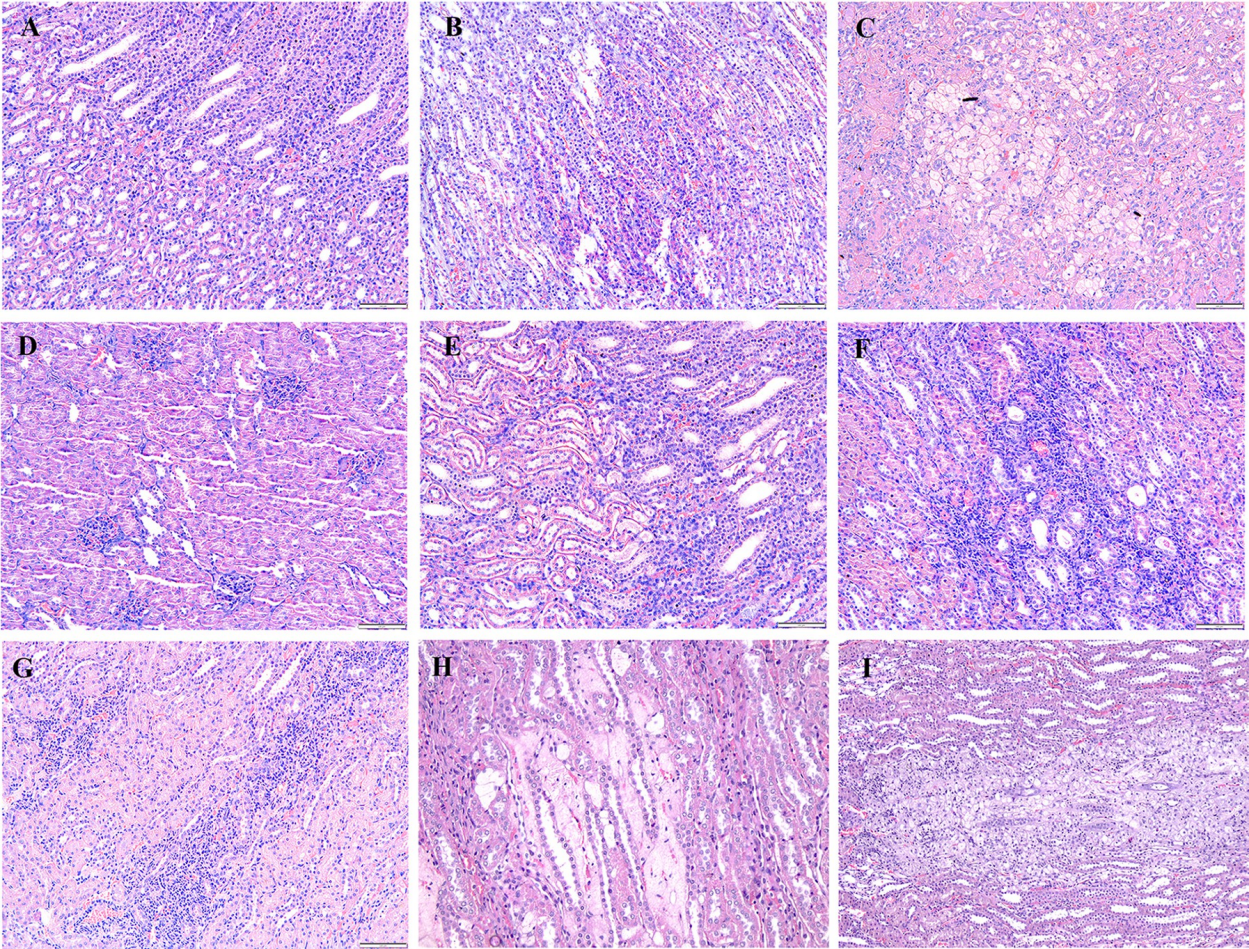
A-I. Photomicrograph of the kidney stained with H&E (10x and 40x). A: Baseline group; B: 4 weeks HCD (arrowhead: mild interstitial inflammation at the medulla); C: 8 weeks HCD (arrow: mild cortical and medullary interstitial inflammation with formation of foam cells); D: Early atherosclerosis group S50; E: Early atherosclerosis group S100; F: Early atherosclerosis group placebo (arrowhead: focal interstitial inflammation at medulla); G: Established atherosclerosis group placebo (arrowhead: Foci of mild interstitial inflammation at the cortico-medullary junction); H: Established atherosclerosis group S50 (arrow: mild inflammation and formation of foam cells in the interstitium); I: Established atherosclerosis group S100 (arrow: mild inflammation and formation of foam cells in the interstitium).

**Fig 11:**
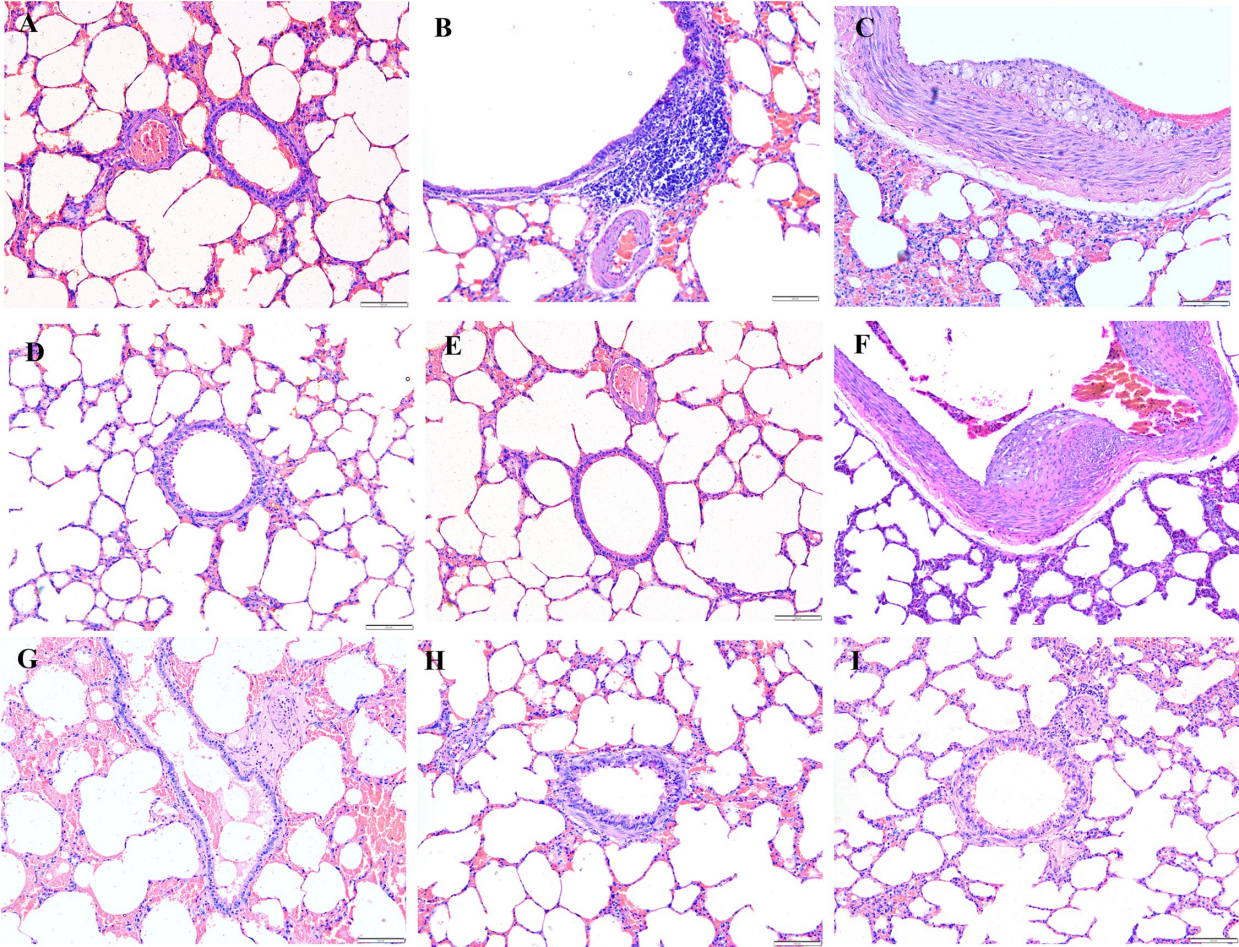
A-I. Photomicrograph of the lungs stained with H&E (10x and 40x). A: Baseline group; B: 4 weeks HCD (arrowhead: mild lymphoid follicles at around bronchiole); C: 8 weeks HCD (arrow: established atherosclerosis of medium sized artery); D: Early atherosclerosis group S50; E: Early atherosclerosis group S100; F: Early atherosclerosis group placebo (arrow: presence of fatty streak in the medium sized artery); G: Established atherosclerosis group placebo (arrow: presence of foam cells in the medium sized artery); H: Established atherosclerosis group S50; I: Established atherosclerosis group S100.

After the rabbits were administered four and eight weeks of HCD, histological examinations indicated abnormalities and toxic effects in the kidneys, liver, heart, brain, and spleen, especially after administering HCD for a longer duration. Intimal edema and the presence of foam cells in one of the medium blood vessels were noticed in the heart (Fig 6A–6I) and perivascular cuffing of lymphocytes was observed in the brain (Fig 7A–7I). The histopathological analysis also revealed the proliferation of hemosiderin-laden macrophages within the red pulp of the spleen (Fig 8A–8I) and mild portal and bridging inflammation, accompanied by steatosis in the liver (Fig 9A–9I). Besides, the renal histopathological analysis showed the presence of mild renal interstitial foam cells (Fig 10A–10I) while the lungs showed intimal thickening of the medium sized blood vessels with presence of foam cells (Fig 11A–11I).

Interestingly, post-treatment with both doses of SEE demonstrated improved results in the pathological examinations of the tissues, as compared to the groups fed HCD and placebo. There was mild interstitial inflammation in the cortico-medullary junction in the kidney and intimal thickening of small and medium blood vessels in the heart. Most of the rabbits in the treated groups demonstrated only a few hemosiderin-laden macrophages in the red pulp of the spleen. Likewise, only a few or no foam cells were detected in all the above-mentioned tissues. In the liver, only mild portal inflammation was observed, with no evidence of bridging inflammation or steatosis. Most of the brain tissues appeared normal; nonetheless, focal areas of perivascular lymphocytic infiltration were observed in some tissues. In the treated rabbit, lung tissues appeared normal. No significant differences were observed in the histology of the tissues between the S50 and S100 groups.

## Discussion

The change in body weight is a crucial sign of toxicity, disease development, and therapeutic response [28]. As expected, HCD caused a significant increase in the body weight of the NZWR in all groups. In this study, NZWR treated with 50 and 100 mg/kg of the SEE depicted a non-significant decrease in body weight compared to the pre-treatment values. This finding is consistent with a previous meta-analysis study in which no significant decrease in body weight was detected after saffron supplementation in obese patients [29]. Besides, an *in vivo* study found that 40 and 80 mg/kg saffron extract reduced body weight, but the result was not significant in male Sprague Dawley rats over 8 weeks of experimental treatment [25]. However, several previous studies demonstrated that ethanolic extract of saffron stigma significantly reduced rats’ body weight [30, 31]. Furthermore, saffron improved the satiety or feeling of fullness and decreased appetite in overweight women, thereby leading to body weight loss [32]. The different durations and doses of saffron stigma ethanolic extract used in the above-mentioned studies might contribute to the discrepancies in the results. In terms of safety, saffron is considered safe as the reduction of body weight in this study is still within the normal range, which is 2.0 to 6.0 kg [33].

In the present study, lipid profile results were analyzed and reported to confirm the development of early and established atherosclerosis in the rabbits, post-intervention with 4 and 8 weeks HCD. LDL and TC increased significantly in all the groups. A higher percentage increase of LDL and TC were observed in rabbits fed with HCD for 8 weeks. This finding corroborates the report from previous studies in which high serum abnormally levels of TC and LDL were associated with an increased risk for atherosclerosis [34, 35].

Hypercholesterolemia is an independent risk factor that can accelerate the development of coronary artery disease and the progression of atherosclerotic lesions [36]. In this study, a significant increase in HDL concentration indicates that HDL may play a protective role by reversing cholesterol transport, inhibiting the oxidation of LDL, and neutralizing the atherogenesis effects of oxidized LDL.

Notably, in early and established atherosclerosis groups, the serum levels of LDL and TC decreased significantly post-treatment with 50 and 100 mg/kg/day SEE. The possible justification for these results is due to the therapeutic effect of saffron. Aligning with the present results, previous studies have demonstrated that saffron exhibits hypolipidemic effects [8, 31, 37, 38].

Toxic agents are known to impair the physiological functions of the liver, kidney, spleen, and other vital organs. Kidneys play a crucial role in the excretion of waste products and toxins, such as urea and creatinine, as well as filtration and reabsorption of the body-needed threshold substance like electrolytes. Thus, the levels of these serum biochemical parameters may be used to assess renal function tests [39]. Such measurements can also reveal the location of cellular tissue damage due to repeated exposure to a potentially toxic agent. The non-clinical safety study recommendations for plant products that have special cause for concern or are intended for a long duration of use may also include an assessment of liver function tests. Following these principles, levels of ALP, ALT, GGT, and AST were measured in the present study and used as liver function tests, while serum urea and creatinine levels were used as renal function tests [40, 41].

In the present study, the serum level of AST significantly increased post-intervention with HCD for eight weeks. In agreement with earlier studies, the elevated ALT and AST levels are attributed to hepatic damage that may contribute to oxidative stress unbalance [42, 43]. This result may indicate that the HCD caused hepatotoxicity by increasing liver enzymes, specifically AST and ALT.

The serum level of ALT was significantly increased compared to pre-treatment in NZWR treated with 100 mg/kg of saffron ethanolic extract. Excess cholesterol intake is a major stimulant for the development of fatty liver [44]. One of the possible reasons for the increase in ALT could be that of fatty liver which is caused by prolonged HCD consumption by the rabbits. Thus, it is postulated that saffron did not have any significant effect in stopping the progression of fatty liver in the established atherosclerotic rabbits. Moreover, a previous study concluded that saffron supplementation could not improve liver function tests including AST, ALT, and ALP [45]. Although saffron increased the level of ALT, it is still within the normal range which is from 25 to 80 U/L, ruling out the toxicity effect of saffron [46, 47].

Several experimental studies on animal models suggested that hyperlipidemia is associated with progressive renal damage. In our study, serum urea significantly increased compared to baseline after administering 1% HCD to the NZWR for 4 to 8 weeks. This result is supported by an earlier study in which mice fed HCD had significantly higher levels of blood urea nitrogen, creatinine, and uric acid compared to the control group [48]. Besides, the result of magnetic resonance imaging analysis in the kidneys of HCD-induced mice revealed renal dysfunction [49]. The increase in serum urea could be due to the consumption of HCD, which generates oxidant load and causes peroxidation, thereby leading to derangements in the rabbit kidney tissue [50].

Serum urea decreased significantly post-treatment with 50 and 100 mg/kg/day SEE in early and established atherosclerosis groups compared to pre-treatment. This result aligns with a previous study, which reported that SEE could decrease blood urea nitrogen and uric acid in type 2 diabetic patients [51]. As a result of SEE supplementation, serum urea levels were reduced, thus leading to a decrease in the renal injury caused by HCD feedings. The present study demonstrated the therapeutic effect of SEE on renal function due to HCD-induced toxicity.

The consumption of a high-fat and high-energy diet is considered a major cause of the development of various complications, such as obesity, cardiovascular and metabolic diseases [52]. As shown in the current study, glucose levels increased in S50 and placebo groups post-intervention with 8 weeks of HCD. The saturated fats present in the high-fat diet are responsible for the increase in glucose and lipid profiles [53]. According to a previous study, rats fed a high-fat diet exhibited an increase in serum glucose concentrations [54].

Blood glucose levels were reduced in NZWR treated with 50 and 100 mg/kg of SEE compared to pretreatment in the early atherosclerosis group and established atherosclerosis groups. These results are supported by previous findings reporting the hypoglycemic properties of saffron [55–58]. The secondary metabolites of saffron, such as flavonoids and terpenes, may be responsible for the present finding [59]. Flavonoids and terpenes have been found to lower blood glucose levels by inhibiting the α-glucosidase enzyme and altering a glucose transporter protein [59, 60].

The role of blood as an index of pathological and physiological status in humans and animals is well-documented [61, 62]. In acute and chronic toxicological studies, changes in hematological and biochemical parameters are usually employed as indices of toxicities. The measurement of RBC counts, HCT and HGB can be used to determine anemia, which could be due to a decrease in the total number of erythrocytes, reduced red blood cell size (MCV), reduced hemoglobin amount per erythrocyte (MCH), or diminished concentration of hemoglobin per total erythrocytes (MCHC) [63].

In the present study, oral administration of 50 and 100mg/kg body weight of SEE for 8 weeks in the early atherosclerosis group elevated the levels of RBC count, HCT, and HGB in NZWR, which might be due to the potential effects of SEE on the activation of erythropoiesis. In contrast to the above effects, the administration of 50 and 100 mg/kg body weight of extract decreased some of the RBC indices, such as MCV and MCH. However, the reductions are still in the normal range which is from 58.5 to 66.5 fL for MCV and 19 to 23 pg for MCH [64].

Neutrophils decreased post-treatment with 4 weeks of administration of HCD, which might stem from the decrease in the adherence of neutrophils to the endothelium during the early responses to injury. A similar result was reported in a previous study after intervention with HCD and a saturated fat diet containing 1% cholesterol plus 1% olive oil for feeding periods of 5, 10, and 15 weeks [65]. Intervention with 4 weeks of HCD administration also revealed a significant elevation of MCH and MCHC compared to baseline, but the values were still in the normal range, which is from 19 to 22.7 pg for MCH and 33 to 50% for MCHC [64]

The increase in lymphocytes post-HCD feeding for 8 weeks could be due to HCD, which interferes with the bone marrow stromal cell–derived factor-1:CXCR4 axis, resulting in lymphocytosis, thrombocytosis, and hematopoietic progenitor cells mobilization [66]. The present study depicted a significant increase in MCH and MCHC post-intervention with 8 weeks of HCD consumption. High MCH scores are commonly a sign of macrocytic anemia while high MCHC indicates that the relative hemoglobin concentration per red blood cell is high [67]. Despite the elevation in the levels of MCH and MCHC, the values were still within the normal range (19 to 22.7 pg for MCH and 33 to 50% for MCHC) [64].

On the contrary, 50mg/kg oral administration of the extract significantly lowered the percentage of MCH and MCHC to their baseline levels. After the administration of 100mg/kg SEE, WBC and lymphocytes markedly decreased, which is consistent with an earlier report [23]. The number of lymphocytes was significantly decreased in response to stressful conditions after the exposure to antigen or extract entrance. Since lymphocytes play key roles in all immune reactions [68], they migrate to sites of inflammations, while their number may concomitantly decrease from the systemic circulation as observed in the present study at the higher dose.

Histopathological studies provide valuable supporting evidence for hematological and biochemical analyses [69]. The photomicrographs of sections of the kidney, liver, heart, brain, and spleen of the rabbits administered HCD for 4 and 8 weeks showed major histological changes, such as interstitial inflammation of the kidney and spleen, intimal edema with the formation of foamy cells in the heart, mild portal, and bridging inflammation with steatosis of the liver and perivascular cuffing of lymphocytes of the brain.

The histopathological analysis presented that 50 and 100mg/kg/day SEE for 8 weeks had improved the damage of tissues in rabbits with early and established atherosclerosis induced by feeding 4 and 8 weeks of HCD, though not in a dose-dependent manner. The observed improvement of the tissues might be attributed to the hypolipidemic effect of SEE, which potentially modulates lipid metabolism and its associated effects. The treatment might have exerted its effects by reducing lipid levels, thereby mitigating lipid-induced damage to the tissues.

### Limitation of the study

This study has potential limitations. This experimental study was conducted in rabbits, and therefore translation on its mechanistic effects on humans must be made with caution. Further clinical trials need to be conducted to confirm the safety of saffron extract on atherosclerotic individuals. Besides, the present study was based on a small sample size since a greater number of animals were not ethically justifiable. However, the sample size was adequate to obtain significant results for all the parameters.

## Conclusions

In conclusion, oral administration of SEE in early and established atherosclerotic NZWR elicited no clinical signs of toxicity or mortality in the evaluated doses administered to the rabbits. Overall, SEE may be classified to be safe, with a broad safety margin for therapeutic use. This study provides valuable data for the safe use of SEE, which should be essential for future pharmacological studies. SEE has a high potential for use in food and drug products, with remarkable benefits for human health. However, further long-term toxicity studies using SEE may be warranted before this extract can be further developed as a nutraceutical or pharmaceutical product.

## Supporting information

S1 File Bodyweight data. https://doi.org/10.6084/m9.figshare.24258571.
(XLSX)

S2 File Lipid profile data. https://doi.org/10.6084/m9.figshare.24258607.
(XLSX)

S3 File Glucose, liver and renal function test data. https://doi.org/10.6084/m9.figshare.24258616.
(XLSX)

S4 File Hematological analysis data. https://doi.org/10.6084/m9.figshare.24258625.
(XLSX)

S5 File Histopathological figures of tissues. https://doi.org/10.6084/m9.figshare.24258643.
(PDF)
