# Correction: Safety evaluation of saffron extracts in early and established atherosclerotic New Zealand white rabbits

**DOI:** 10.1371/journal.pone.0298279

**Published:** 2024-01-31

**Authors:** Iman Nabilah Abd Rahim, Noor Alicezah Mohd Kasim, Effat Omar, Suhaila Abdul Muid, Hapizah Nawawi

There are errors in the Funding statement and Acknowledgements.

The correct Funding statement is as follows: This research was funded by the Ministry of Higher Education Malaysia through the Fundamental Research Grant Scheme (FRGS/1/2019/SKK06/UITM/03/4) and Geran Insentif Penyeliaan Universiti Teknologi MARA (UiTM) (600-RMC/GIP 5/3 (067/2023)) which were awarded to the corresponding author (NAMK). The URLs of the funders are https://www.mohe.gov.my/en and https://rmc.uitm.edu.my/index.php/research-funding/national-grant respectively. The funders had no role in study design, data collection and analysis, decision to publish, or preparation of the manuscript.

The correct Acknowledgements are as follows: The authors would like to acknowledge the Ministry of Higher Education Malaysia for funding this project through the Fundamental Research Grant Scheme (FRGS) (FRGS/1/2019/SKK06/UITM/03/4) and Universiti Teknologi MARA (UiTM) for funding this project through Geran Insentif Penyeliaan UiTM (600-RMC/GIP 5/3 (067/2023)). Additionally, we would like to thank the Institute of Medical Molecular Biotechnology, Laboratory Animal Care Unit, and Clinical Diagnostic Laboratories of the Faculty of Medicine, UiTM for providing the facilities to complete this study.
