# Supplementary material for: Safety evaluation of saffron extracts in early and established atherosclerotic New Zealand white rabbits
Source: PLoS One. 2024 Jan 11;19(1):e0295212. doi: 10.1371/journal.pone.0295212 (PMC10783933; doi:10.1371/journal.pone.0295212)
Supplement: S5 File — https://doi.org/10.6084/m9.figshare.24258643. (PDF) [file pone.0295212.s005.pdf]

LIVER

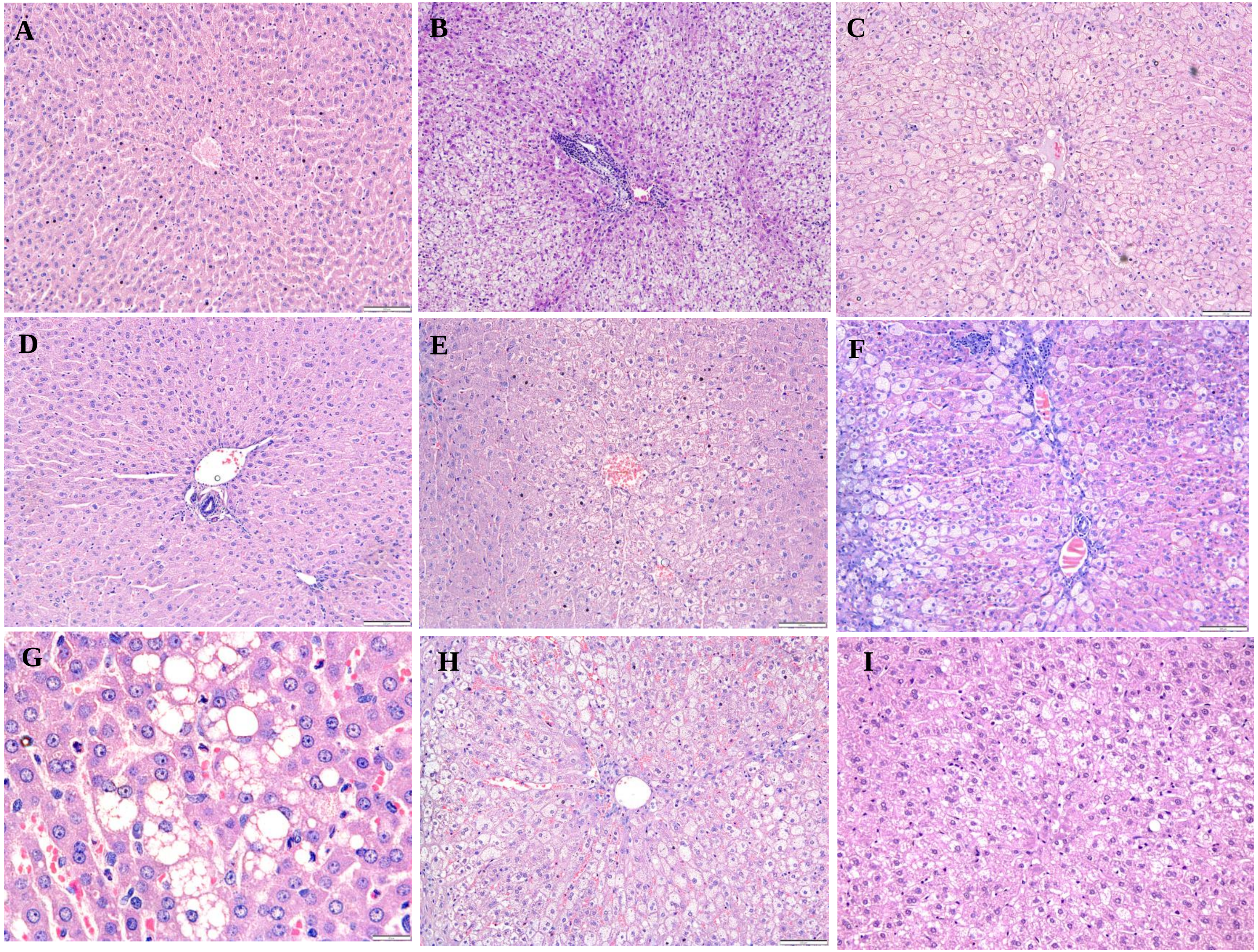

A: Normal diet
B: 4 weeks HCD
C: 8 weeks HCD
D: Early S50
E: Early s100
F: Early placebo
G: Est Placebo
H: EST S50
I: EST S100

KIDNEY

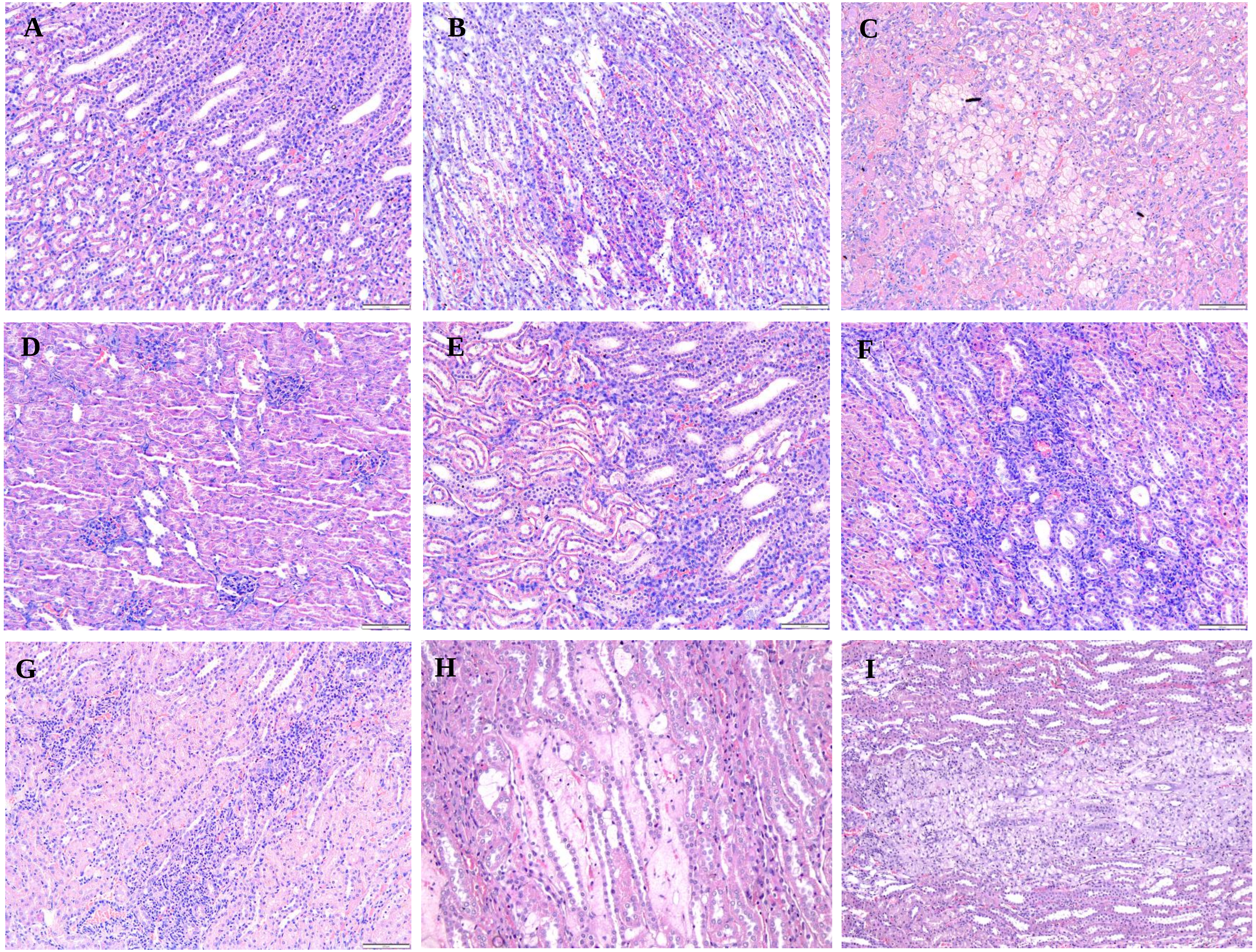

A: Normal diet
B: 4 weeks HCD
C: 8 weeks HCD
D: Early S50
E: Early s100
F: est placebo
G: Placebo
H: EST S50
I: EST S100

BRAIN

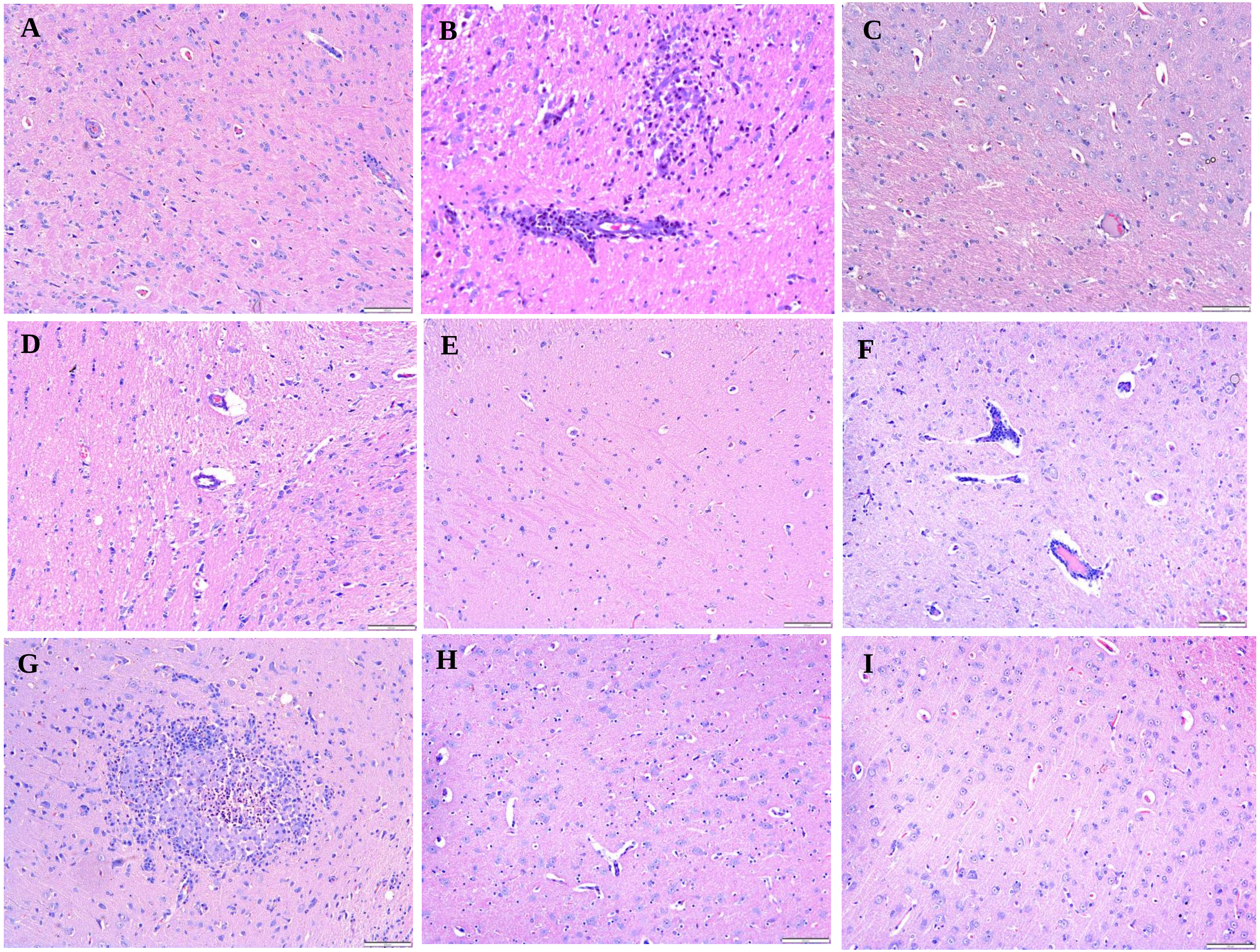

A: Normal diet
B: 4 weeks HCD
C: 8 weeks HCD
D: Early S50
E: Early s100
F: est placebo
G: Placebo
H: EST S50
I: EST S100

LUNGS

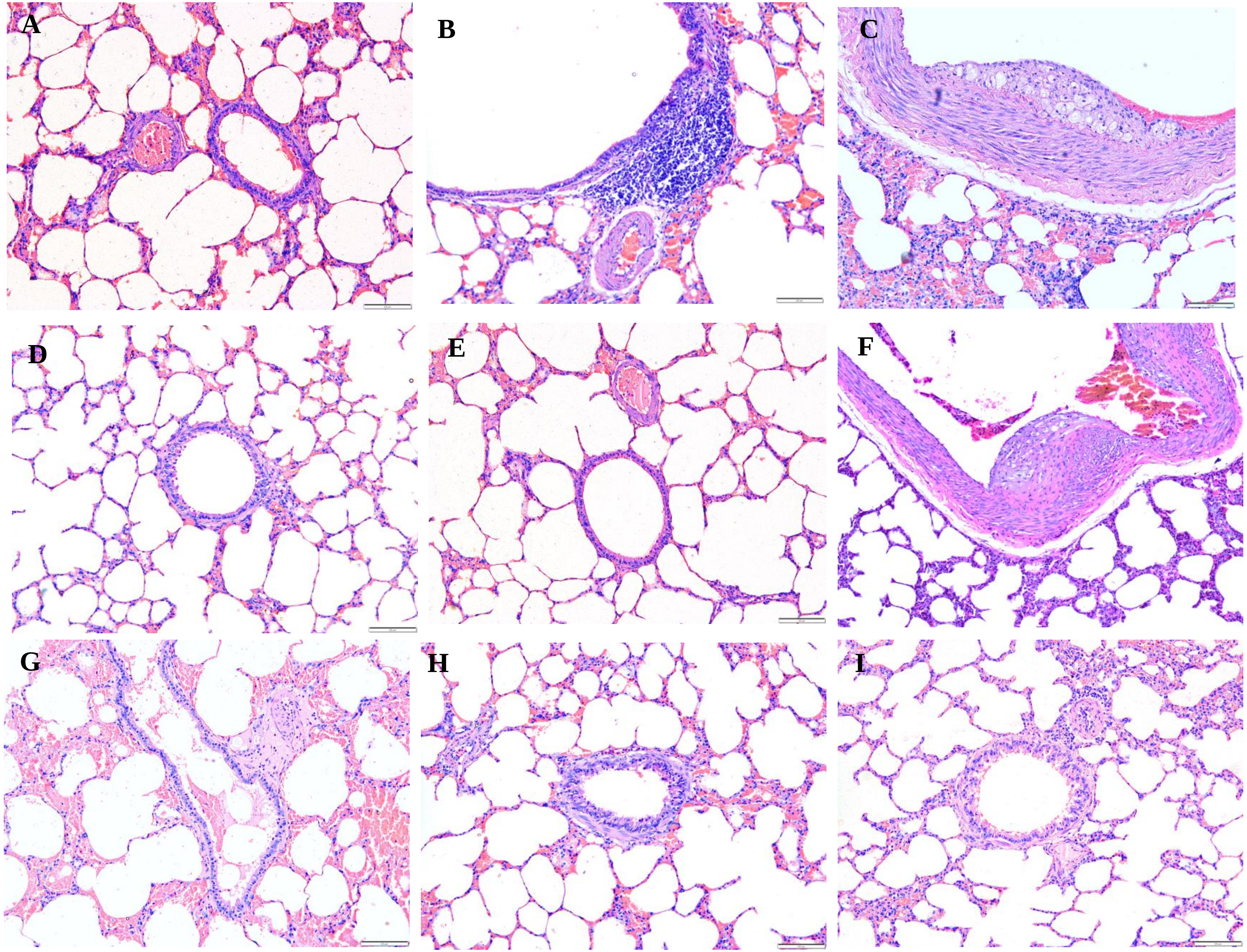

A: Normal diet
B: 4 weeks HCD
C: 8 weeks HCD
D: Early S50
E: Early s100
F: est placebo
G: Placebo
H: EST S50
I: EST S100

HEART

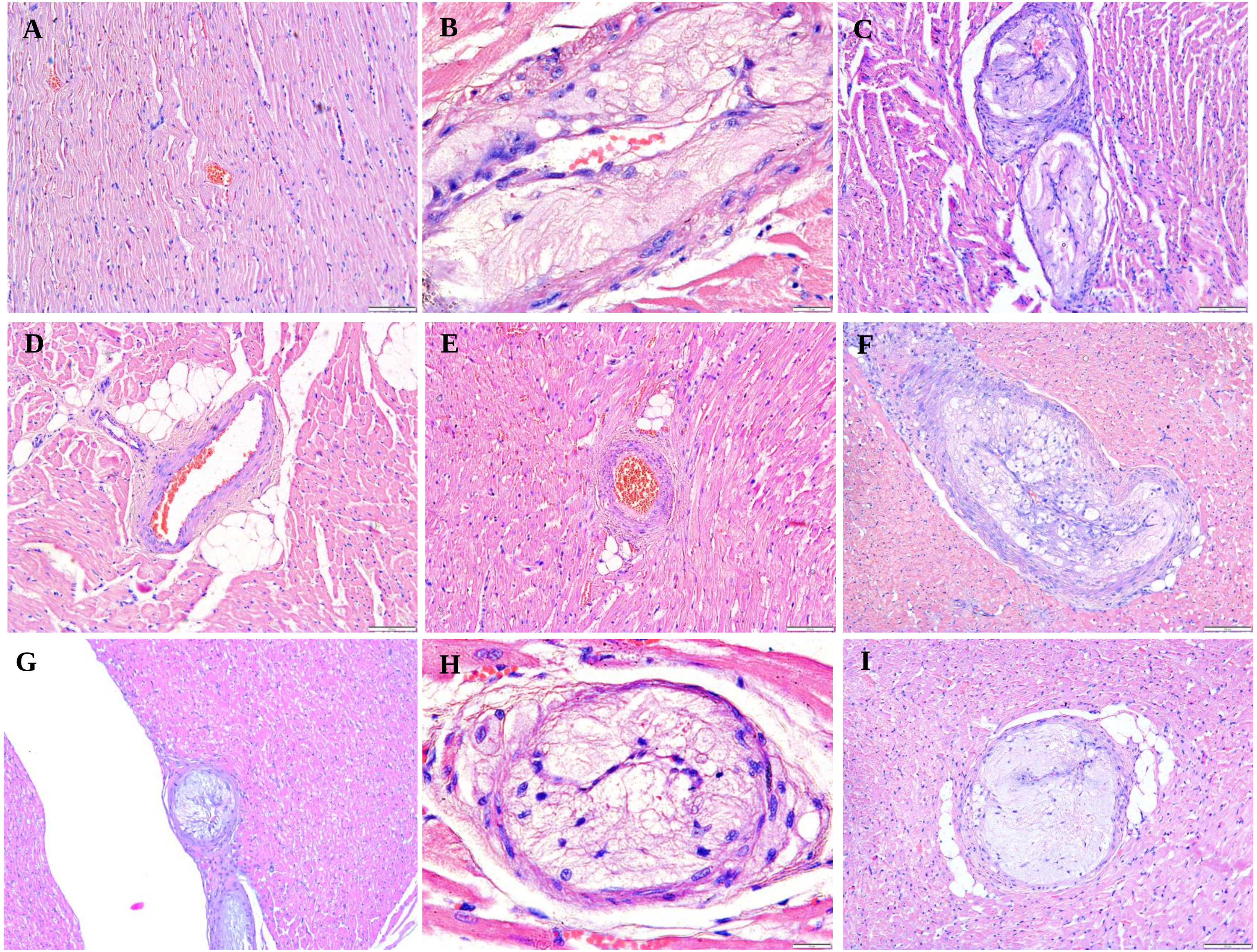

A: Normal diet
B: 4 weeks HCD
C: 8 weeks HCD
D: Early S50
E: Early s100
F: est placebo
G: Placebo
H: EST S50
I: EST S100

SPLEEN

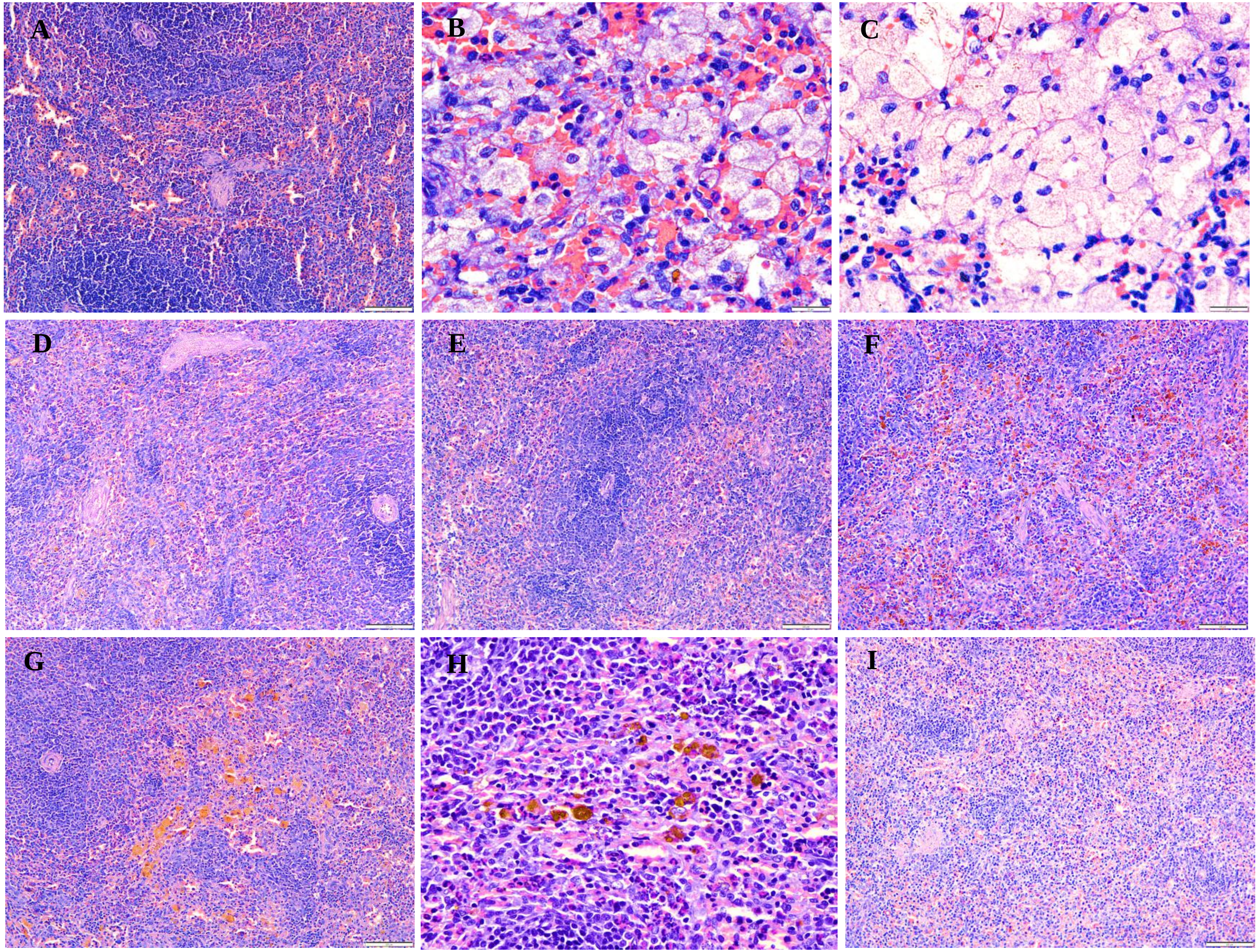

A: Normal diet
B: 4 weeks HCD
C: 8 weeks HCD
D: Early S50
E: Early s100
F: est placebo
G: Placebo
H: EST S50
I: EST S100
